# Computational Approaches to tRNA-Derived Small RNAs

**DOI:** 10.3390/ncrna3010002

**Published:** 2017-01-04

**Authors:** Wei-Lin Xu, Ye Yang, Yi-Dan Wang, Liang-Hu Qu, Ling-Ling Zheng

**Affiliations:** Key Laboratory of Gene Engineering of the Ministry of Education, State Key Laboratory of Biocontrol, School of Life Sciences, Sun Yat-sen University, Guangzhou 510275, China; xuwl6@mail2.sysu.edu.cn (W.-L.X.); yangy238@mail2.sysu.edu.cn (Y.Y.); wangyd8@mail2.sysu.edu.cn (Y.-D.W.)

**Keywords:** next-generation sequencing, tRNA-derived small RNA, tRNA halves, tRNA-derived small RNA fragments, identification, database

## Abstract

tRNA-derived small RNAs (tDRs) are a group of small, non-coding RNAs derived from transfer RNAs (tRNAs). They can be classified as tRNA halves and tRNA-derived small RNA fragments (tRFs). Accumulating experimental evidence suggests their functional roles in cells and in various biological processes. Advances in next-generation sequencing (NGS) techniques allow a large amount of small RNA deep-sequencing data to be generated. To investigate tDRs from these data, software to identify tDRs and databases to retrieve or manage tDR data have been devised. In this review, we summarized the tools and databases for tDR identification and collection, with the aim of helping researchers choose the best tools for their analysis and inspiring the invention or improvement of tools in the field.

## 1. Introduction

The last decade witnessed the discovery of an enormous number of tRNA-derived small RNAs (tDRs) in almost all branches of life [1,2,3,4,5,6,7,8,9,10,11,12]. It has been suggested that these tDRs are not products of the random degradation of transfer RNAs (tRNAs), but are generated by precise processes. Accumulating experimental evidence suggests that they have important regulatory roles in translation, viral infections and tumor development [13,14,15,16,17,18].

tDRs are classified as tRNA halves and tRNA-derived small RNA fragments (tRFs). tRNA halves are generated by a single cleavage at the anticodon of mature tRNAs, and their lengths range from 30 nt to 35 nt [1]. The biogenesis of tRNA halves is mostly induced by stress, such as oxidative stress [1]. tRFs, however, are generated from both precursor (pre-tRNAs) and mature tRNAs through Dicer-dependent or -independent processes, and are approximately 20 nt in size. However, this classification of tDRs is not definite. The features used to classify tRNA halves and tDRs are not unambiguous, and their functions and mechanisms overlap.

Early studies investigated tDRs individually and revealed that they are highly expressed in cells [19]. Recently, deep sequencing tools have been developed to comprehensively detect tDRs in silico [20]. The discovery of tDRs has raised the need for the storage and management of massive amounts of tDR data. Recent tDR identification tools and databases are mostly designed for eukaryotic cells. Consequently, in this review, we focus only on eukaryotic tDRs, and discuss the computational approaches and tools for identifying and managing the data for eukaryotic tDRs.

## 2. tDRs

In eukaryotic cells, tRNA genes are transcribed by RNA polymerase III, and the resulting products (precursor tRNAs or pre-tRNAs) undergo further processing before maturation (mature tRNAs). These processes remove the 5’ end (5’ leader, by RNase P) and the 3’ end (3’ trailer, by RNase Z) of pre-tRNAs and then add the 5’-CCA-3’ (CCA) trinucleotide to the 3’ end of the tRNAs after cleavage. The splicing of tRNA transcripts and the extensive chemical modification to generate non-canonical bases also occur with these processes [21,22,23].

tDRs can be roughly classified as tRNA halves and tRFs. tRNA halves are formed by cleavage at the anticodon of mature tRNAs. Rny1p, a member of the RNase T2 family, and angiogenin, a member of the RNase A superfamily, are responsible for the cleavage to produce tRNA halves in yeast and mammalian cells, respectively [13,24,25]. Rny1p and angiogenin possess little substrate specificity. Rny1p is contained within the vacuole of the yeast cells and is released into the cytoplasm during stress-induced situations [26,27]. Angiogenin is secreted and endocytosed into the cytoplasm [28]. Typically, these enzymes are inhibited, and stress induces their release from the inhibitors [29].

Two groups of tRNA halves have been discovered, namely 5’ halves and 3’ halves [29]. Besides, sex hormone-dependent tRNA-derived RNAs (SHOT-RNAs) were found to be constitutively expressed in human breast and prostate cancers [17]. They were considered tRNA halves, share counterparts of 5’ and 3’ halves, and possess biogenesis factors similar to those of tRNA halves. However, the expression of SHOT-RNAs is induced by sex hormones, and they are generated from tRNA species different from those of tRNA halves [17]. Diverse functional mechanisms of tRNA halves were suggested. It has been shown that endogenous 5’ tRNA halves inhibit translation through the displacement of elF4G/A (eukaryotic translation initiation factors) from the mRNA transcripts [13,30]. Thompson and Parker proposed four possible functional models of tRNA halves: the inhibition of translation through nicked tRNAs, the formation of a repression complex with other unknown proteins, guiding the inhibition of translation through their association with Argonaute or Piwi, and guiding the cleavage of mRNAs via their interaction with the tRNA processing enzymes RNase Z or RNase P [29].

**Figure 1:**
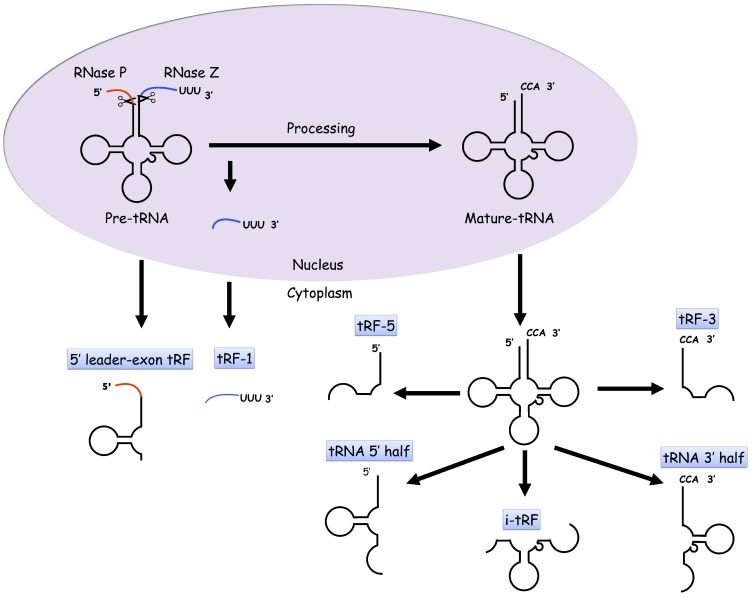
Biogenesis of tRNA-derived small RNAs (tDRs). In eukaryotic cells, precursor tRNAs undergo processing and become mature tRNAs. The cleavage of precursor tRNAs generates tRNA-derived small RNA fragments (tRF-1s) and 5’ leader-exon tRFs. tRF-5s, tRF-3s, i-tRFs, 5’ halves and 3’ halves are generated by the cleavage of mature tRNAs.

tRFs, however, are produced from pre-tRNAs as well as mature tRNAs. Up until now, four types of tRFs have been identified and characterized by their provenance on tRNAs: tRF-1s, tRF-5s, tRF-3s, and i-tRFs. tRF-1s result from the cleavage of 3’ trailer fragments of precursor tRNAs by RNases, including Dicer and RNase Z [11,19,31], and they usually begin exactly after the 3’-ends of mature tRNAs (5’-CCA-3’ tails excluded) and possess poly-Us at their 3’-ends [19]. tRF-5s are generated by cleavage in the D-loop of tRNAs by Dicer, oftentimes with adenine as their 3’-ends [19,31]. tRF-3s result from cleavage in the T-loop by Dicer, angiogenin and other members of the RNase A superfamily and are fragments of the 3’ end of mature tRNAs (with CCA at the 3’ end); the cleavage usually occurs between A/U and A/U near the 3’-end of the tRNAs [19,30,31]. i-tRFs are enriched within the internal regions of mature tRNAs, and they usually straddle the anticodon and begin right after the second or downstream nucleotide of the 5’-ends of tRNAs [32]. Recently, a previously undescribed 5’ leader-exon tRNA fragment type was discovered to be associated with the loss of spinal motor neurons in CLP1-kinase dead mice [30,33]. These fragments straddle the 5’ leader sequence of pre-tRNAs and the 5’ end of mature tRNAs. The mechanism of the tRF regulation of gene expression remains elusive. tRFs were shown to associate with Argonautes similar to siRNAs and miRNAs and were thus assumed to silence gene expression [31,34]. It has also been shown that the displacement of pro-oncogenic transcripts with tRFs from the RNA-binding protein YBX1 might account for the suppression of human breast cancer progression by the tRFs [18]. In addition, the tRNA 3’ external transcribed spacers might be excised and function as a sponge to reduce noise in transcription [35]. Figure 1 shows the general pathways for tDR biogenesis.

The functions of tDRs are not well understood. It was found that tDRs are correlated to the differentiation, development and metabolism of primitive eukaryotes [36,37]. In the mouse, the mature sperm enrichment of tDRs was discovered, and the tDRs were thought to act as epigenetic factors or as regulators of endogenous retro-elements prior to implantation of the embryo [10,38,39]. In humans, tDRs have been found in several human cancer cell lines, but their correlation to tumorigenesis and progression remains elusive [16,17,18,19,40,41,42]. Compared with those in animals, the tDRs in plants are less investigated. tDRs were found to accumulate in the roots of *Arabidopsis thaliana* or in the shoots of barley during phosphate deprivation [43,44]. A more recent study revealed the existence of conserved tDRs in *Arabidopsis thaliana*, *Oryza sativa* and *Physcomitrella patens* [45].

## 3. Identifying tDRs and Managing tDR Data

Research on tDRs requires tools that facilitate the identification or retrieval of information on tRFs. In general, studies on tDRs could be classified into three categories:

- identifying the tDRs;
- analyzing tDR functions;
- storing or managing and manipulating data.

Computational approaches aimed at assisting researchers with these steps arose, and the following is a general review of the tools and databases for tDRs.

### 3.1. Identifying tDRs

In this review, three types of computational solutions to tDR identification will be summarized: general tools not specific to tDR identification in early studies, the command-line pipelines designed for tDR identification, and the integrated web server tRF2Cancer with user-friendly interfaces.

The main goal of small RNA deep-sequencing data analysis is to find the source types of RNAs and quantify their expression. Therefore, the mapping of reads is an important step. Early studies used BLAST [16,19,37,46,47], bowtie/bowtie2 [48,49], exonerate [40], and the self-made string matching alignment algorithm to map small RNAs from the NGS data and identify tDRs from them [50].

Recent studies began to raise concerns regarding the subtleties of mapping to the genome versus to the tRNA alone. These concerns arose from the proposal of two different strategies to detect tDRs in 2015: the strategy proposed by Telonis et al., and the tDRmapper developed by Selitsky and Sethupathy. Telonis et al. proposed to identify candidate tRNA-fragment reads by mapping them to the genome, whereas the tDRmapper maps the trimmed reads directly to tRNAs [32,50]. Telonis et al. mapped the reads to the whole genome and sought those reads with exact matches only to the tRNA sequences. In particular, they allowed for only exact matches in mapping. They claimed that with the limited lengths of tDRs and the sequence similarities among copies of tRNAs of the same anticodon, and even of different anticodons specifying the same amino acids, the allowance for mismatches might possibly confound the origins of tDRs. With regard to the requirements of exact matches, the three nucleotides immediately downstream of the 3’ ends of tRNAs are replaced by CCA prior to mapping, to reserve CCA-trinucleotide-tail containing reads. Additionally, they permitted multiple hits on genomic tRNAs and retained the reads spanning exon–exon junctions but not those stepping partially on the introns. In their study, they identified a fourth class of tRFs, i-tRFs, and encouraged scepticism to the rationality of mapping the reads to the tRNA space alone in tRF identification. However, this study did not provide off-the-shelf software [32]. Almost at the same time, Selitsky and Sethupathy released tDRmapper, a command-line tool for the identification, naming and quantification of tDRs [50]. Of particular note is that tDRmapper adopted an “error type” hierarchical alignment scheme to handle possible mismatches attributed to chemical modifications. In each step, tDRmapper takes the reads that were unmapped in the last step as inputs. The inputs are then aligned with pre-tRNAs or tRNAs with some defined requirements, such as exact matches with mature or pre-tRNAs, as one to two mismatches or one to three base pair deletions with mature tRNAs. The resulting mappable reads in each step are collected, annotated and quantified as candidate tRFs.

The striking difference between the strategy of Telonis et al. and tDRmapper is whether or not the reads are mapped to the reference genome. In tDRmapper, the incomplete annotations of tRNA genes in the genome and the computational capacity are mentioned as reasons for not mapping to the genome. Specifically, reads mappable to both tRNAs and other regions in the genome are possibly indicative of new unannotated tRNA loci but are not reads derived from genomic templates other than tRNAs [50]. In contrast, Telonis et al. proposed that, due to tRNA lookalikes of nuclear and mitochondrial tRNAs and partial tRNA sequences, the reads mappable both to tRNAs and other known non-tRNA sequences should be excluded because their origins are dubious. Accounting for the number of loci in the genome with lengths and sequences similar to known tRNAs, the mapping to tRNAs alone would generate higher false positives and exaggerate the expression of certain tRFs from given tRNAs [32,50]. Thus, all these controversies can be reduced to the balance between obtaining false positive and false negative results [51]. tDRmapper aimed at identifying more potential tRFs at the cost of increased false positives, whereas the Telonis study aimed at providing more accurate tRFs and excluded reads that were unlikely to derive from tRNAs.

To strike a balance between the total inclusion and total exclusion of dubious reads, we recently designed tRF2Cancer, an integrated web server for identifying tRFs from small RNA sequencing data and evaluating their expression in cancers [48]. tRF2Cancer consists of tRFfinder (tRF identification from small RNA deep-sequencing reads), tRFinCancer (inspect expression of tRFs across cancer types) and tRFBrowser (origins and chemical modifications of tRFs). Prior to mapping, tRFfinder maps the processed reads to the human genome and human transcript sequences to remove exogenous reads and reads other than tRFs (such as mRNAs, miRNAs, snoRNAs, snRNAs, rRNAs and repeats). This step helps to reduce the false positive results derived from non-tRF reads. tRFfinder then maps the reads to tRNA sequences to reduce false negative results. To evaluate the enrichment of reads on tRNAs after mapping, a binomial test was introduced. Although mismatches and indels are allowed in mapping by making use of bowtie/bowtie2, tRFfinder is cautious, and a scoring scheme was introduced. In this scheme, reads with mismatches or indels are given lower scores than those with exact matches, and mismatches resulting from chemical modifications are thus distinguished by way of differing scores. tRFfinder is the first web-based, user-friendly tool for the identification of tRFs. It strikes a balance between the total inclusion and total exclusion of dubious reads by giving reads of sceptical origins a lower score. tRFfinder is designed specifically for identifying human tRFs and their expression in multiple types of cancers. At the current stage, it only provides the web-based version for researchers to upload their sequencing data to the server; the command-line version is under development. Table 1 shows the comparison between the work of Telonis et al., tDRmapper and tRFfinder.

To conclude, identifying tRFs from random degradation will remain a challenge. In addition to probability-based methods for evaluating the enrichment of reads on the genome or tRNAs, machine-learning algorithms might prove promising. In microRNA identification, machine-learning-based methods have been adopted to identify microRNAs based on known sequences and structural properties [52]. In addition, the lengths or cleavage site preferences are features that might be used in machine-learning-based methods to identify tDRs.

### 3.2. Databases of tDRs

Databases of tRFs help researchers obtain tRF sequences and evaluate expression counts in multiple experiments [47]. However, only a limited number of tRF databases are available in the field. In this review, four databases are introduced: tRFdb, tRFinCancer (in the tRF2Cancer), MINTbase, and the Olvedy et al. repertoire of prostate cancer.

tRFdb is a relational database of tRFs and other tRNA-related RNA fragments [47]. tRFdb contains tRF records from eight species ranging from bacteria to human. The database provides a uniform nomenclature and unique tRF ID for the tRFs. tRFdb provides information on tRF (original organisms, type, source tRNAs, sequence) and samples that contain the corresponding tRF reads, with interfaces to the GEO (Gene Expression Omnibus) and SRA (Sequence Read Archive) database. In addition, tRFdb displays the distribution of reads on tRNA sequences. To construct the database, tRFdb mapped the reads from the GEO/SRA database to the tRNA sequences, and the reads that were enriched in the tRNA sequences were further mapped to the whole genomes to exclude those reads mappable to outside tRNAs. The database contains tRF records from eight species, and therefore, it is suitable for research on tRF evolution or comparisons among species. However, the database contains only records of tRF-5s, tRF-3s, tRF-1s; it contains no records of i-tRFs or other tDRs. Furthermore, all the records in the database are results of bioinformatic prediction and require further experimental validation.

tRFinCancer is a database for viewing the expression of tRFs in multiple cancer types [48]. In tRFinCancer, the tRFs are predicted from TCGA (The Cancer Genome Atlas) data that contain the small RNA deep-sequencing data of 32 human cancer types and subtypes. The tRFs are identified using the tRFfinder. One feature of tRFinCancer is that users can determine the expression of tRFs in 32 cancer types; through comparisons of the expression in multiple cancers, researchers might be able to spot correlations between cancers and tRF expression levels. We hope that tRFinCancer can provide a starting point for functional studies of tRFs and their correlation with cancers.

tRFs can be generated from both nuclear tRNAs as well as mitochondrial tRNAs [53]. MINTbase is a database for tRFs of mitochondrial and nuclear origin [54]. In MINTbase, users can access five types of tDRs: tRF-5s, tRF-3s, i-tRFs, 5’-halves and 3’-halves of mitochondrial and nuclear origin. Different from the probability-based search methods used by tRFdb, MINTbase adopted the deterministic and exhaustive approach to search for all possible tRF candidates and retain those fragments mappable exclusively to tRNAs. In MINTbase, two methods are provided to users to view tRFs. Users can select and view tRFs according to tRF types, precursor tRNAs, and the precursor tRNA anticodon. Users can also search tRFs by tRNA names, fragment sequence, and by fragment label (defined by MINTbase). In addition to ordinary information on tRFs (i.e., sequence information, expression information, parental tRNA information and genomic information), based on the results page returned by MINTbase, users can view tRFs by five so-termed “vistas”: genomic loci, RNA molecule, tRNA alignment, expression and summary. The two most striking features of MINTbase are that (1) it provides all-inclusive information on exhaustive tRF species of nuclear or mitochondrial origin and that (2) it provides interfaces for the submission of new tRF records. The ability to submit new records is important for updating a database. Currently, all the tRF records in MINTbase are produced from bioinformatic prediction and need to be validated by experiments in the future.

Olvedy et al. designed a repertoire of tRFs that focused exclusively on prostate cancer [55]. In the study, they examined and identified differentially expressed tRFs and their compositions in clinical prostate cancer samples representing different stages of the cancer. They also quantified the expression of tRFs across samples. The study provides a comprehensive catalogue (termed “database”) of tRFs in different stages of prostate cancer; however, it does not provide a user-friendly interface.

As mentioned, tRFdb and MINTbase contain almost all tDR records in a given species, but do not provide a more focused view of tDRs in certain diseases. tRFinCancer offers options to view the expression of a given tDR across cancer types, which makes comparisons among cancers convenient; however, it does not provide a general overview of all tDRs in a given disease. The Olvedy et al. repertoire, however, is helpful for users to view the expression of tDRs in a given disease, namely prostate cancer. Therefore, the Olvedy et al. repertoire complemented by tRFinCancer might be helpful to researchers in that they offer a comprehensive view of tDR expression and their correlation to diseases. A comparison between the databases is shown in Table 2.

## 4. Perspectives

tDRs are experiencing intense research, and their biological functions are gradually being revealed. Currently, off-the-shelf computational tools for identifying these small RNAs and databases to manage their information are available. However, as an integrated field of tDRs, many other areas of study are in need of tools for the analysis of tDRs. Three notable fields are the prediction of targets of tDRs, the study of their correlation to diseases, and the study of circulating tDRs. Currently, there are no tools for target prediction of tDRs. In retrospect, CLIP-Seq (cross-linking and Argonaute immunoprecipitation coupled with high-throughput sequencing) can be used to search for microRNA targets utilizing the microRNA:mRNA interaction mediated by Argonaute (AGO) proteins [56,57,58], and databases for these data are present (for instance, starBase [59,60]). Similar techniques have been applied to study tDR:RNA interactions; for instance, meta-analysis of data generated by PAR-CLIP (photoactivatable-ribonucleoside-enhanced crosslinking and immunoprecipitation), CLASH (crosslinking, ligation, and sequencing of hybrids) and HITS-CLIP (high-throughput sequencing of RNA isolated by crosslinking immunoprecipitation, also referred to as CLIP-Seq) revealed the tRF association preferences for AGOs and mRNAs [46,61]. These are promising methods to unravel the interactive dynamics between tDRs and their target proteins or RNAs. Tools to analyze and manage these data can facilitate the exploration of tDR biological functions by providing information on the targets of tDRs.

Insufficient tools are in place for the study of the correlations between tDRs and diseases. In retrospect, there are two dimensions in the study of correlations between tDRs and some given diseases. The first dimension highlights that given a sample from a disease of interest, the expression of different tDRs in this sample is detected to assess the up- or down-regulation of tDRs and to determine their expression patterns [16,19,40,41,42]. This dimension gives the overall patterns of tDR expression in the disease and offers some indications on the interactive dynamics between tDRs. This dimension of study requires pipelines and databases that focus on the disease under study, and Olvedy et al.’s repertoire is one example of this type of database [55]. The raw deep-sequencing data and analysis results provided by the tDR studies in different diseases, and the use of bioinformatic pipelines to predict tDRs from small RNA deep-sequencing data from samples of different diseases, are resources to build this type of database. The second dimension highlights the cross-comparison of tDR expression profiles in different types of diseases that can be used to study whether up- or down-regulation of the given tDRs is common to all related diseases or is unique to one specific case. This dimension indicates the potential of tDRs as diagnostic biomarkers in disease detection. One example of this type of database is tRFinCancer, which enables users to compare the expression of the tDR of interest in multiple cancers [48]. The first dimension lacks databases or computational pipelines that focus on the diseases under study, and the second dimension requires more inclusive databases. More work is required to tailor the pipelines or databases on the tDR for adequate study of diseases.

Accumulating evidence suggests the presence of circulating, functional tDRs in mouse serum [62,63], dairy cows [63,64], and humans [25,63,65]. The functions of circulating tDRs are not well characterized, and their potential as diagnostic biomarkers remains to be elucidated. In view of this, the databases to collect tDRs from circulating small RNA fragments from normal or disease samples, by bioinformatic prediction or collection of results from previous studies, might be helpful to the study of circulating tDRs.

In addition to new requirements from novel fields of study, several problems in the recent development of pipelines and databases remain. To systematically study tDRs and facilitate communication in the literature, it is important to establish a standard nomenclature for tDRs. However, the nomenclatures of tDRs are inconsistent among the different tools and different databases [48,50,53]. The difficulties of establishing a standardized tRF nomenclature lie in that only a small fraction of tDRs are validated by experiments. In addition, the origin of tDRs cannot be deterministically traced to one particular precursor tRNA due to tRNA isotypes; some common sequence motifs are shared among tRNAs with different anticodons coding for the same amino acid, and even allowing for one mismatch/insertion/deletion would probably confound the origin of tDRs. Thus, before establishing a uniform nomenclature for tDR, more research is needed to identify methods to trace tDRs back to their tRNA precursors.

Chemical modifications on tRNAs affect the RT-PCR amplification of tDRs and thus the false positive rates of tools in the field. Extensive chemical modifications of the small RNAs would abort the reverse transcription in RT-PCR and thus generate truncated reads whose 3’ ends overlap exactly with these sites [66,67]. Additionally, reverse transcriptase might pause at the chemically modified sites, which would enhance the likelihood of inserting the wrong nucleotides at these sites. Therefore, chemical modifications of the tRNAs and the corresponding tDRs might lead to increased truncated reads and higher chances of mismatch. However, recent computational tools to identify tDRs rarely take into account the effect of chemical modifications. To reduce the effect of truncated reads on identification, in tRFfinder we discarded the prediction results of tDRs whose 3’ ends overlap with the chemically modified sites [48]. It is more difficult to address the problem of mismatch from chemical modifications. tRFfinder introduces the scoring matrix to distinguish the sources of mismatches. If the mismatches occur at the sites of chemical modification, 0.5 point per site is subtracted from the total score, and 1 point per site otherwise [48].

Telonis et al. prevented mismatch from mapping reads to the genome, indicating that even one mismatch might confound the origin of tDRs [32,50]. This might introduce high false negative rates, since mismatches resulting from chemical modifications prevent the true tDR reads from mapping. However, tDRmapper allowed mismatches according to a hierarchical alignment scheme, which might confound mismatches of different origins. To address this contradiction, mismatches of different origins should be set apart, and in tRFfinder, a scoring scheme could help [48]. There are also new sequencing methods to handle the effect of chemical modifications. For instance, the AlkB-facilitated RNA methylation sequencing (ARM-Seq) method treats the samples with de-alkylating enzymes to remove methylations from tRNAs before RT-PCR [66]. This way, the effect of chemical modifications can be eliminated at the beginning, and RT-PCR can provide more accurate reads representative of small RNAs.

Nowadays, computational tDR tools mainly focus on identification and databases. The introduction of more reliable computational and statistical methods, or novel methods based on machine learning, is desired to accurately identify tDRs from small RNA sequencing data. Besides, more specialized databases are also expected to satisfy the diverse requirements of the researchers. In addition, new fields of study on tDRs are growing, while the tools are lacking in these fields. So the development of novel computational methods for tDR research will be a challenging but fruitful endeavour.

## Figures and Tables

**Table 1:** List of tools specific to RNA-derived small RNA (tDR) identification.

Name	Mapping to	Potential Effects	Mapping Tools	Mismatches, Indels, and Chemical Modification	tDR Types Detectable	User Interface
Telonis et al., [32,50]	Genome to exclude reads mappable outside tRNAs	Potential false negative	–	Mismatches/indels not allowed	All four tRF types	No packaged pipelines provided
tDRmapper	tRNAs alone	Potential false positive	Built-in algorithms	Error type hierarchy	Multiple tRFs and tRNA halves	Command line
tRFfinder	First to genome and known transcripts, then to tRNAs	Moderate false positive & negative	bowtie/bowtie2	Allowed (with options), but given different weights (scores) in result page	Multiple tDR including all tRF types	Website interface

**Table 2:** List of databases of tDRs.

Name	Records	Methods	Species	User Interface
tRFdb	tRF-3s, -5s, -1s from nucleus	Bioinformatic prediction	Eight species	A website to search and view tRFs
tRFinCancer	All four types from nucleus across cancer types	Bioinformatic prediction	32 human cancer types	A website to view tRFs expression across cancers
MINTbase	All types from mature, nuclear and mitochondrial tRNAs	Bioinformatic prediction	Human	Website to search and view tRFs
Olvedy et al. repertoire	All types in prostate cancer	Bioinformatic prediction, with qPCR quantification to validate differential expression of selected tRFs	Human prostate cancer	A catalogue

## References

[1] Lee S.R., Collins K. (2005). Starvation-induced cleavage of the tRNA anticodon loop in *Tetrahymena thermophila*. J. Biol. Chem..

[2] Zheng L.L., Wen Y.Z., Yang J.H., Liao J.Y., Shao P., Xu H., Zhou H., Wen J.Z., Lun Z.R., Ayala F.J. (2013). Comparative transcriptome analysis of small noncoding RNAs in different stages of *Trypanosoma brucei*. RNA.

[3] Gebetsberger J., Zywicki M., Künzi A., Polacek N. (2012). tRNA-derived fragments target the ribosome and function as regulatory non-coding RNA in *Haloferax volcanii*. Archaea.

[4] Heyer R., Dörr M., Jellen-Ritter A., Späth B., Babski J., Jaschinski K., Soppa J., Marchfelder A. (2012). High throughput sequencing reveals a plethora of small RNAs including tRNA derived fragments in *Haloferax volcanii*. RNA Biol..

[5] Nunes C.C., Gowda M., Sailsbery J., Xue M., Chen F., Brown D.E., Oh Y., Mitchell T.K., Dean R.A. (2011). Diverse and tissue-enriched small RNAs in the plant pathogenic fungus, *Magnaporthe oryzae*. BMC Genom..

[6] Åsman A.K., Vetukuri R.R., Jahan S.N., Fogelqvist J., Corcoran P., Avrova A.O., Whisson S.C., Dixelius C. (2014). Fragmentation of tRNA in *Phytophthora infestans* asexual life cycle stages and during host plant infection. BMC Microbiol..

[7] Couvillion M.T., Bounova G., Purdom E., Speed T.P., Collins K. (2012). A *Tetrahymena Piwi* bound to mature tRNA 3’ fragments activates the exonuclease Xrn2 for RNA processing in the nucleus. Mol. Cell.

[8] Soares A.R., Fernandes N., Reverendo M., Araújo H.R., Oliveira J.L., Moura G.M., Santos M.A. (2015). Conserved and highly expressed tRNA derived fragments in zebrafish. BMC Mol. Biol..

[9] Karaiskos S., Naqvi A.S., Swanson K.E., Grigoriev A. (2015). Age-driven modulation of tRNA-derived fragments in *Drosophila* and their potential targets. Biol. Direct.

[10] Peng H., Shi J., Zhang Y., Zhang H., Liao S., Li W., Lei L., Han C., Ning L., Cao Y. (2012). A novel class of tRNA-derived small RNAs extremely enriched in mature mouse sperm. Cell Res..

[11] Babiarz J.E., Ruby J.G., Wang Y., Bartel D.P., Blelloch R. (2008). Mouse ES cells express endogenous shRNAs, siRNAs, and other Microprocessor-independent, Dicer-dependent small RNAs. Genes Dev..

[12] Chen C.J., Liu Q., Zhang Y.C., Qu L.H., Chen Y.Q., Gautheret D. (2011). Genome-wide discovery and analysis of microRNAs and other small RNAs from rice embryogenic callus. RNA Biol..

[13] Ivanov P., Emara M.M., Villen J., Gygi S.P., Anderson P. (2011). Angiogenin-induced tRNA fragments inhibit translation initiation. Mol. Cell.

[14] Wang Q., Lee I., Ren J., Ajay S.S., Lee Y.S., Bao X. (2013). Identification and functional characterization of tRNA-derived RNA fragments (tRFs) in respiratory syncytial virus infection. Mol. Ther..

[15] Selitsky S.R., Baran-Gale J., Honda M., Yamane D., Masaki T., Fannin E.E., Guerra B., Shirasaki T., Shimakami T., Kaneko S. (2015). Small tRNA-derived RNAs are increased and more abundant than microRNAs in chronic hepatitis B and C. Sci. Rep..

[16] Maute R.L., Schneider C., Sumazin P., Holmes A., Califano A., Basso K., Dalla-Favera R. (2013). tRNA-derived microRNA modulates proliferation and the DNA damage response and is down-regulated in B cell lymphoma. Proc. Natl. Acad. Sci. USA.

[17] Honda S., Loher P., Shigematsu M., Palazzo J.P., Suzuki R., Imoto I., Rigoutsos I., Kirino Y. (2015). Sex hormone-dependent tRNA halves enhance cell proliferation in breast and prostate cancers. Proc. Natl. Acad. Sci. USA.

[18] Goodarzi H., Liu X., Nguyen H.C., Zhang S., Fish L., Tavazoie S.F. (2015). Endogenous tRNA-derived fragments suppress breast cancer progression via YBX1 displacement. Cell.

[19] Lee Y.S., Shibata Y., Malhotra A., Dutta A. (2009). A novel class of small RNAs: tRNA-derived RNA fragments (tRFs). Genes Dev..

[20] Friedländer M.R., Mackowiak S.D., Li N., Chen W., Rajewsky N. (2012). miRDeep2 accurately identifies known and hundreds of novel microRNA genes in seven animal clades. Nucleic Acids Res..

[21] Abbott J.A., Francklyn C.S., Robey-Bond S.M. (2014). Transfer RNA and human disease. Molecular Biology of the Transfer RNA Revisited.

[22] Hopper A.K., Phizicky E.M. (2003). tRNA transfers to the limelight. Genes Dev..

[23] Sobala A., Hutvagner G. (2011). Transfer RNA-derived fragments: Origins, processing, and functions. Wiley Interdiscip. Rev. RNA.

[24] Thompson D.M., Parker R. (2009). The RNase Rny1p cleaves tRNAs and promotes cell death during oxidative stress in Saccharomyces cerevisiae. J. Cell Biol..

[25] Fu H., Feng J., Liu Q., Sun F., Tie Y., Zhu J., Xing R., Sun Z., Zheng X. (2009). Stress induces tRNA cleavage by angiogenin in mammalian cells. FEBS Lett..

[26] MacIntosh G.C., Bariola P.A., Newbigin E., Green P.J. (2001). Characterization of Rny1, the Saccharomyces cerevisiae member of the T2 RNase family of RNases: Unexpected functions for ancient enzymes?. Proc. Natl. Acad. Sci. USA.

[27] Thompson D.M., Lu C., Green P.J., Parker R. (2008). tRNA cleavage is a conserved response to oxidative stress in eukaryotes. RNA.

[28] Moroianu J., Riordan J.F. (1994). Nuclear translocation of angiogenin in proliferating endothelial cells is essential to its angiogenic activity. Proc. Natl. Acad. Sci. USA.

[29] Thompson D.M., Parker R. (2009). Stressing out over tRNA cleavage. Cell.

[30] Huang H.Y., Hopper A.K. (2016). Multiple layers of stress-induced regulation in tRNA biology. Life.

[31] Haussecker D., Huang Y., Lau A., Parameswaran P., Fire A.Z., Kay M.A. (2010). Human tRNA-derived small RNAs in the global regulation of RNA silencing. RNA.

[32] Telonis A.G., Loher P., Honda S., Jing Y., Palazzo J., Kirino Y., Rigoutsos I. (2015). Dissecting tRNA-derived fragment complexities using personalized transcriptomes reveals novel fragment classes and unexpected dependencies. Oncotarget.

[33] Hanada T., Weitzer S., Mair B., Bernreuther C., Wainger B.J., Ichida J., Hanada R., Orthofer M., Cronin S.J., Komnenovic V. (2013). CLP1 links tRNA metabolism to progressive motor-neuron loss. Nature.

[34] Loss-Morais G., Waterhouse P.M., Margis R. (2013). Description of plant tRNA-derived RNA fragments (tRFs) associated with argonaute and identification of their putative targets. Biol. Direct.

[35] Lalaouna D., Carrier M.C., Semsey S., Brouard J.S., Wang J., Wade J.T., Massé E. (2015). A 3’ external transcribed spacer in a tRNA transcript acts as a sponge for small RNAs to prevent transcriptional noise. Mol. Cell.

[36] Liao J.Y., Guo Y.H., Zheng L.L., Li Y., Xu W.L., Zhang Y.C., Zhou H., Lun Z.R., Ayala F.J., Qu L.H. (2014). Both endo-siRNAs and tRNA-derived small RNAs are involved in the differentiation of primitive eukaryote Giardia lamblia. Proc. Natl. Acad. Sci. USA.

[37] Li Y., Luo J., Zhou H., Liao J.Y., Ma L.M., Chen Y.Q., Qu L.H. (2008). Stress-induced tRNA-derived RNAs: A novel class of small RNAs in the primitive eukaryote Giardia lamblia. Nucleic Acids Res..

[38] Chen Q., Yan M., Cao Z., Li X., Zhang Y., Shi J., Feng G.H., Peng H., Zhang X., Zhang Y. (2016). Sperm tsRNAs contribute to intergenerational inheritance of an acquired metabolic disorder. Science.

[39] Sharma U., Conine C.C., Shea J.M., Boskovic A., Derr A.G., Bing X.Y., Belleannee C., Kucukural A., Serra R.W., Sun F. (2016). Biogenesis and function of tRNA fragments during sperm maturation and fertilization in mammals. Science.

[40] Kawaji H., Nakamura M., Takahashi Y., Sandelin A., Katayama S., Fukuda S., Daub C.O., Kai C., Kawai J., Yasuda J. (2008). Hidden layers of human small RNAs. BMC Genom..

[41] Cole C., Sobala A., Lu C., Thatcher S.R., Bowman A., Brown J.W., Green P.J., Barton G.J., Hutvagner G. (2009). Filtering of deep sequencing data reveals the existence of abundant Dicer-dependent small RNAs derived from tRNAs. RNA.

[42] Liao J.Y., Ma L.M., Guo Y.H., Zhang Y.C., Zhou H., Shao P., Chen Y.Q., Qu L.H. (2010). Deep sequencing of human nuclear and cytoplasmic small RNAs reveals an unexpectedly complex subcellular distribution of miRNAs and tRNA 3’ trailers. PLoS ONE.

[43] Hsieh L.C., Lin S.I., Shih A.C.C., Chen J.W., Lin W.Y., Tseng C.Y., Li W.H., Chiou T.J. (2009). Uncovering small RNA-mediated responses to phosphate deficiency in Arabidopsis by deep sequencing. Plant Physiol..

[44] Hackenberg M., Huang P.J., Huang C.Y., Shi B.J., Gustafson P., Langridge P. (2013). A comprehensive expression profile of microRNAs and other classes of non-coding small RNAs in barley under phosphorous-deficient and-sufficient conditions. DNA Res..

[45] Alves C.S., Vicentini R., Duarte G.T., Pinoti V.F., Vincentz M., Nogueira F.T. (2016). Genome-wide identification and characterization of tRNA-derived RNA fragments in land plants. Plant Mol. Biol..

[46] Kumar P., Anaya J., Mudunuri S.B., Dutta A. (2014). Meta-analysis of tRNA derived RNA fragments reveals that they are evolutionarily conserved and associate with AGO proteins to recognize specific RNA targets. BMC Biol..

[47] Kumar P., Mudunuri S.B., Anaya J., Dutta A. (2015). tRFdb: A database for transfer RNA fragments. Nucleic Acids Res..

[48] Zheng L.L., Xu W.L., Liu S., Sun W.J., Li J.H., Wu J., Yang J.H., Qu L.H. (2016). tRF2Cancer: A web server to detect tRNA-derived small RNA fragments (tRFs) and their expression in multiple cancers. Nucleic Acids Res..

[49] Singleton C.K., Delude R.L., Ken R., Manning S.S., McPherson C.E. (1991). Structure, expression, and regulation of members of the developmentally controlled V and H gene classes from Dictyostelium. Dev. Genet..

[50] Selitsky S.R., Sethupathy P. (2015). tDRmapper: Challenges and solutions to mapping, naming, and quantifying tRNA-derived RNAs from human small RNA-sequencing data. BMC Bioinform..

[51] Telonis A.G., Loher P., Kirino Y., Rigoutsos I. (2016). Consequential considerations when mapping tRNA fragments. BMC Bioinform..

[52] Li L., Xu J., Yang D., Tan X., Wang H. (2010). Computational approaches for microRNA studies: A review. Mamm. Genome.

[53] Telonis A.G., Kirino Y., Rigoutsos I. (2015). Mitochondrial tRNA-lookalikes in nuclear chromosomes: Could they be functional?. RNA Biol..

[54] Pliatsika V., Loher P., Telonis A.G., Rigoutsos I. (2016). two types of: a framework for the interactive exploration of mitochondrial and nuclear tRNA fragments. Bioinformatics..

[55] Olvedy M., Scaravilli M., Hoogstrate Y., Visakorpi T., Jenster G., Martens-Uzunova E. (2016). A comprehensive repertoire of tRNA-derived fragments in prostate cancer. Oncotarget.

[56] Chi S.W., Zang J.B., Mele A., Darnell R.B. (2009). Argonaute HITS-CLIP decodes microRNA-mRNA interaction maps. Nature.

[57] Zisoulis D.G., Lovci M.T., Wilbert M.L., Hutt K.R., Liang T.Y., Pasquinelli A.E., Yeo G.W. (2010). Comprehensive discovery of endogenous Argonaute binding sites in Caenorhabditis elegans. Nat. Struct. Mol. Biol..

[58] Hafner M., Landthaler M., Burger L., Khorshid M., Hausser J., Berninger P., Rothballer A., Ascano M., Jungkamp A.C., Munschauer M. (2010). Transcriptome-wide identification of RNA-binding protein and microRNA target sites by PAR-CLIP. Cell.

[59] Yang J.H., Li J.H., Shao P., Zhou H., Chen Y.Q., Qu L.H. (2011). starBase: A database for exploring microRNA-mRNA interaction maps from Argonaute CLIP-Seq and Degradome-Seq data. Nucleic Acids Res..

[60] Li J.H., Liu S., Zhou H., Qu L.H., Yang J.H. (2014). starBase v2. 0: Decoding miRNA-ceRNA, miRNA-ncRNA and protein-RNA interaction networks from large-scale CLIP-Seq data. Nucleic Acids Res..

[61] Pillai M.M., Gillen A.E., Yamamoto T.M., Kline E., Brown J., Flory K., Hesselberth J.R., Kabos P. (2014). HITS-CLIP reveals key regulators of nuclear receptor signaling in breast cancer. Breast Cancer Res. Treat..

[62] Dhahbi J.M., Spindler S.R., Atamna H., Yamakawa A., Boffelli D., Mote P., Martin D.I. (2013). 5’ tRNA halves are present as abundant complexes in serum, concentrated in blood cells, and modulated by aging and calorie restriction. BMC Genom..

[63] Dhahbi J.M. (2014). Circulating small noncoding RNAs as biomarkers of aging. Ageing Res. Rev..

[64] Casas E., Cai G., Neill J.D. (2015). Characterization of circulating transfer RNA-derived RNA fragments in cattle. Front. Genet..

[65] Akat K.M., Moore-McGriff D., Morozov P., Brown M., Gogakos T., Da Rosa J.C., Mihailovic A., Sauer M., Ji R., Ramarathnam A. (2014). Comparative RNA-sequencing analysis of myocardial and circulating small RNAs in human heart failure and their utility as biomarkers. Proc. Natl. Acad. Sci. USA.

[66] Cozen A.E., Quartley E., Holmes A.D., Hrabeta-Robinson E., Phizicky E.M., Lowe T.M. (2015). ARM-seq: AlkB-facilitated RNA methylation sequencing reveals a complex landscape of modified tRNA fragments. Nat. Methods.

[67] Motorin Y., Muller S., Behm-Ansmant I., Branlant C. (2007). Identification of modified residues in RNAs by reverse transcription-based methods. Methods Enzymol..

